# Perinatal Risk Factors and Genu Valgum Conducive to the Onset of Growing Pains in Early Childhood

**DOI:** 10.3390/children3040034

**Published:** 2016-11-18

**Authors:** Angelos Kaspiris, Efstathios Chronopoulos, Elias Vasiliadis

**Affiliations:** 1 Department of Trauma and Orthopaedics, Thriasio General Hospital—NHS, G. Gennimata av, Magoula 19600, Athens, Greece; 2 Second Department of Orthopaedic Surgery, Konstantopoulio General Hospital and Medical School, University of Athens, Athens 14233, Greece; stathi24@yahoo.gr; 3 Third Department of Orthopaedic Surgery, KAT General Hospital and Medical School, University of Athens, Kifissia 14561, Athens, Greece; eliasvasiliadis@yahoo.gr

**Keywords:** growing pains, genu valgum, perinatal factors, bone metabolism

## Abstract

The most prevalent musculoskeletal disorder of childhood with unclear aetiology is growing pains (GPs). Anatomic deformities and factors that change bone turnover are implicated in GP pathophysiology. Perinatal risk factors alter the bone metabolism affecting the bone mineral density and content. The aim of our study was to analyze the relationship between GPs, knock knees and perinatal factors. The examined population consisted of 276 children aged 3–7 years. Among them, ten pairs of dizygotic twins were evaluated. The data were collected by using a combination of semi-structured questionnaires, clinical examinations and medical charts of the children and the obstetric history of the mothers. A total of 78 children presenting GPs met Peterson’s criteria. Genu valgum severity was a significant factor for GP manifestation and for their increased frequency and intensity. Subsequently, perinatal factors regarding gestational age, Apgar score, head circumference (lower than 33 cm) and birth length or weight (smaller than 50 cm and 3000 g, respectively) made a remarkable contribution to the development of GPs. Conversely, antenatal corticosteroid treatment, increased maternal age and maternal smoking during pregnancy were not predictive of the disorder. Our data are potentially supportive for the “bone strength” theory and for the contribution of anatomical disturbances in GP appearance.

## 1. Introduction

Growing pains (GPs) are the most common form of episodic musculoskeletal pains during childhood. GPs mainly affect children between the ages of 3–12 years [1,2,3,4], but their highest frequency is detected in the age-group of 4–6 years [3]. Although GPs were first described by the French physician Duchamp in 1823 [5], their underlying pathophysiological mechanism still remains an enigma. In order to explain their development, various theories have been supported. The first theory was anatomical and centered on the observation that lower limb deformities (flat-feet or valgus knee) induce the appearance of pains [6]. Despite the fact that this theory has been recently weakened, it is still under investigation [2]. The second theory is the nocturnally accelerated bone theory [7] that analyzes the association between increased growth spurts and the appearance of GPs. However, there are no validated findings confirming that bone growth can be reason for pain [2]. Other theories include the local overuse syndrome theory which remains untested [8], the non-inflammatory pain syndrome theory [9], which is supported by reports for increased activity levels of children in more than 40% of the publications [2], and the hypothesis of early childhood’s pain amplification syndrome [10]. Furthermore, a twin family survey [11] provided evidence that GPs appear to bear genetic susceptibility and present genetic association with restless leg syndrome. However, as there is no reliable and valid assessment tool testing hypermobility in children, the last hypothesis remains unproven [2]. Currently, many studies focus on the correlation of growing pains with bone density [8] and with factors affecting paediatric bone metabolism regarding serum levels of vitamin D or calcium [8] and omega-3 fatty acid intake [12,13,14,15,16]. The bone strength theory, which is closely related to the non-inflammatory pain syndrome theory, is based on the observation that the tibial bone density in children with GPs, as it was measured by quantitative ultrasound, was significantly reduced compared to healthy children [8]. Furthermore, the serum levels of vitamin D3 in GPs patients were remarkably low [12,13,15,16]. Similarly, in the affected children, the bone mineral status of the phalanx was low and accompanied by increased serum levels of parathyroid hormone (PTH), whereas the alkaline phosphatase levels were normal [12]. However, the bone strength theory is still subject to further research.

It is recognized from animal-based and clinical studies that perinatal risk factors including gestational age [17,18], birth length [18,19], birth weight [19,20] and maternal smoking or ethanol consumption [21] as well as the antenatal use of corticosteroids [18,22,23,24,25], contribute towards alterations in a newborn’s bone metabolism having a direct impact on the mineralization of osteoid tissue during infancy. As the skeletal development is strongly related with bone changes during fetal and newborn periods, evaluation of the factors influencing mineral metabolism during early childhood can lead to useful conclusions about skeletal functionality in later life.

The aim of this study is to analyze the possible association between the development and intensity of GPs with perinatal characteristics in children 3–7 years old. Moreover, this research examines the possible interaction between the severity of valgus knee deformity and perinatal risk factors in the development of GPs in children.

## 2. Materials and Methods

### 2.1. Study Design

This is a retrospective study that was conducted based on the combination of semi-structured questionnaires (a mixture of open and closed questions), clinical examinations and clinical data collected by the medical charts of the children and the obstetric history of the mothers. Particularly, our methodological approach was as follows: First we selected and evaluated the GP cases based on Peterson’s criteria and afterwards we reviewed the medical charts for children’s perinatal characteristics. It took place from January 2014 to January 2015 during the children’s visits to the paediatric department of the co-operated National Primary Care Trust. These visits took place for routine check-ups and vaccinations. Overall, 276 children aged 3–7 years old, including ten pairs of dizygotic twins, were examined on a daily basis. To determine the correct sample size for our survey, Cochran’s formula was used: *n = Z^2^pq/e^2^*, where *Z* is the confidence level (1.96), *p* is the variability (0.50), *q* (0.50) and *e* is the precision (7%). Our sample size was 30% larger than the minimum number of participants (*n* = 196) required to ensure accurate results based on the above parameters. The sample was also representative of sex, social economic status and geographical distribution, including children from local urban, suburban and rural areas, as it was based on the social insurance of the participants.

The children’s parents or guardians were fully informed about the nature and the aim of the study and written consent was obtained (reference number of National’s Primary Health Care Board approval: 3305/01-6-2013). The study complies with the Helsinki Declaration of 1964.

### 2.2. Inclusion and Exclusion Criteria

The inclusion criteria of our research were based on Peterson’s [26] definition standards for GPs. These are as follows: recurrent pains in both lower extremities, with duration of up to 72 h, usually in the late afternoon or evening, with no arthritic localization or impaired joint mobility, in the absence of rubor, oedema, sensitivity, lesions, infections or other disorders. Exclusion criteria were history of known bone disease or musculoskeletal disorders such as fractures or chronic inflammation, clinical signs of articular disease including swelling, redness, reduced joint range, or trauma. Additionally, children with bone metabolism diseases (i.e., chronic renal syndrome, osteogenesis imperfecta, diastrophic dwarfism) or being under treatment with drugs that increase bone metabolism (i.e., glucocorticoids, anticonvulsants, antiretroviral therapy) were excluded from the study.

### 2.3. The Questionnaire

The questionnaire used included questions concerning the appearance of GPs during the year before the children visit our department, their frequency, and information about their age, sex, ethnicity, medical or orthopaedic history and medication. The questionnaire of the study was developed by the validated survey of Evans and Scutter [27]. The Wong-Baker FACES Pain Rating Scale (FACES scale) was used in order to assess pain intensity. The FACES scale is displayed on a horizontal line of six hand-drawn faces that ranged from a smiling face on the left to a crying face on the right, being scored from zero to ten respectively. We use this scale because its accuracy during the self-report pain intensity in children [28] that develop GPs [12] is well documented.

The zygosity of the twin pairs was assessed after applying the 12-question formula concerning the general similarity between twins, as it was introduced by Jackson et al. [29,30].

Finally, the height and weight of the children were measured and their body mass index (BMI) was calculated. A BMI within the 5th and 95th sex-specific and age-specific percentiles using the Center for Disease Control and Prevention (CDC) revised growth charts was considered normal.

### 2.4. Tibiofemoral Angle and Intermalleolar Distance

The tibiofemoral (TF) angle and the intermalleolar (IM) distance were measured with a clinical method introduced by Cheng et al. [31] that was also used by our team as thoroughly described in a previous study [32]. Specifically, the lower limbs were carefully positioned during the assessment. The children were made to stand, ensuring full extension and neutral rotation at the hips and knees, with the knees or ankles touching each other closely. The superior iliac spines, the region and the centre of the knee and the midpoint of the ankle joint were marked with a dermographic pen. Subsequently, the TF angle was measured very carefully in both legs. The valgus angle was measured with a positive sign. The distance between the malleoli was measured in an upright position using a tape measure and was also expressed with a positive sign. In assessing the IM distance, the knees were pressed at the medial condyles of both limbs against each other. This was helpful in the measurement of obese children with fat thighs [32]. The examination was performed by an experienced paediatric orthopaedic surgeon.

### 2.5. Perinatal Factors

The perinatal characteristics were based on the medical charts of the children and the maternal obstetrical history. Specifically, we retrieved information regarding gestational age, birth weight, length, head circumference, Apgar score, maternal infection, mode of delivery and use of medication, alcohol or smoking during pregnancy. Gestational age had been estimated from the first day of the last menstrual period. Children with birth weight between the 10th and 90th percentiles for gestational age were deemed appropriate for gestational age.

Furthermore, data were retrieved for the antenatal use of corticosteroids from the obstetrical files concerning the gestational age at administration of corticosteroids and the number of corticosteroid courses. A complete course of corticosteroid therapy was defined as at least one course of therapy 24 h before delivery [33].

### 2.6. Statistical Analysis

The data were initially entered on a Microsoft Excel 2007 (Microsoft, Redmond, WA, USA) spreadsheet and then analyzed statistically using SPSS version 21.0 (IBM Corporation, New York, NY, USA). Initially, the data were summarized using descriptive statistics (mean, standard deviation (SD) and 95% confidence interval). Subsequently, the normality of the variables was studied using the Kolmogorov-Smirnov and the Shapiro-Wilk tests. Additionally, Levene’s test was used to assess the equality of variances. Where the distribution of variables was normal, the Student’s *t*-test was used, whereas in cases of divergence in the distribution of a variable, we proceeded with the Mann-Whitney U-test. The contribution of the independent variables to the development of GPs was also studied using logistic regression analysis. Due to the limited number of dizygotic twins, the non-parametric Wilcoxon signed-rank test was used. Pearson chi-square test was also used. The level of statistical significance was set to 5% (*p* = 0.05).

## 3. Results

### 3.1. Growing Pains and Demographic Characteristics

A total of 261 Caucasian children (124 boys and 137 girls) were eligible for inclusion in the study, whereas 15 children were excluded. There were 78 children (49 boys and 29 girls) who met the criteria of Peterson [25] for GPs (prevalence = 29.9%). The children’s demographics are shown in Table 1.

### 3.2. GPs and Tibiofemoral Angle/Intermalleolar Distance

The mean values, SDs, Min/Max and confidence interval for the anatomic femoral-tibial angles and for the IM distance in both groups are presented in Table 2. Increased degree of genu valgum was positively correlated with the development of GPs in children (Table 2). Specifically, TF angle and IM distance are statistically associated with the appearance of GPs (*p* < 0.01 and *p* < 0.01, respectively).

### 3.3. GPs and Perinatal Factors

Statistical analysis revealed that perinatal risk factors are positively correlated with the development of GPs during childhood. In particular, a short gestation period (*p* < 0.01), low Apgar score (*p* < 0.02)and small birth length (*p* < 0.01), or birth weight (*p* < 0.01) and head circumference (*p* < 0.01) (Table 2) increase the risk of GPs. Interestingly, the risk of GP development increased when the birth weight of the child was lower than 3000 g. According to the study’s data (Table 3) 36 out of 187 (19.3%) children suffering from GPs weighed over 3000 g at birth, while 42 out of 74 (56.8%) weighed less than 3000 g (*p* < 0.01). Similarly (Table 3), the birth head circumference in 53 out of 219 (24.2%) children suffering from GPs was greater than 33 cm while in 25 out of 42 (59.5%) was less than 33 cm (*p* < 0.01). On the contrary, maternal age (*p* = 0.91), maternal smoking during pregnancy (*p* = 0.05) and the antenatal use of corticosteroids (*p* = 0.57) does not seem to be implicated in the development of GPs (Table 2 and Table 3).

Conclusively, the logistic regression analysis confirms that male sex, children’s increased age, severity of genu valgum and gestational age of the affected children are statistically correlated with the appearance of GPs (Table 4). On the contrary, low Apgar score, increased maternal age and smoke exposure are not statistically related with GP development.

### 3.4. Types, Frequency and Intensity of GPs

We also studied the impact of genu valgum severity and perinatal risk factors on the form and frequency of GPs. As shown in Table 4, IM distance and maternal age are not statistically associated with the type of GP (Table 5).

On the contrary, the perinatal characteristics including small birth length, head circumference and weight are strongly correlated with GPs (Table 4 and Table 5). In particular, birth length smaller than 50 cm, birth weight lower than 3000 g and head circumference less than 33 cm are statistically correlated with nocturnal waking, crying and pains in both legs (Table 6).

Furthermore, the weekly appearance of GPs (Table 6 and Table 7) is statistically linked with reduced head circumference (*p* < 0.01) and birth weight (*p* < 0.01), though it is not associated with IM distance (*p* = 0.86), birth length (*p* = 0.19) or maternal age (*p* = 0.45). We must underline that although the antenatal administration of corticosteroids was not a contributing factor to the development of GPs, the frequency and the intensity of GPs were elevated in the children who had been exposed to dexamethasone (Table 6 and Table 8).

Pain intensity was examined using the FACES scale (Table 9). The statistical analysis revealed a correlation between GP severity and gestational age (*p* < 0.01), birth length (*p* < 0.01), birth weight (*p* < 0.01) and head circumference (*p* < 0.01).

### 3.5. GPs in Dizygotic Twins

In our study, ten pairs of dizygotic twins were included and all of the above-mentioned variables were examined. Under the assumption that twin pairs share, to a significant extent, environmental and genetic influences, we aim to test if anatomic differences are related with the appearance of GPs. Interestingly, the appearance of GPs in the twins was not affected by the anthropometric characteristics including current weight, height or BMI nor the perinatal factors (Table 10). Contrariwise, elevated TF angle and IM distance (Table 10) are positively associated with the appearance of GPs (*p* = 0.02 and *p* = 0.02, respectively).

## 4. Discussion

The most common cause of musculoskeletal pain during childhood are GPs, which remain largely inexplicable. Despite the fact that GPs are widely considered a benign syndrome and their resolution occurs during late childhood, they may have deleterious effects leading many researchers to explore their underlying pathophysiological mechanism. To the best of our knowledge, this is the first study that investigates the association of children’s perinatal characteristics with the appearance of GPs and examines the correlation of genu valgum severity using clinical methodology, with the frequency and intensity of GPs. However, our research has a number of limitations. First, it is retrospective and the data rely on the information accuracy of the interviewed children or parents. Moreover, inherited design limitations appear in retrospective studies such as inability to collect missing or unreported data, parental difficulties in recalling information and the studies not being blind. However, they allow accumulation of data for a significant number of patients, thus minimizing possible errors. Secondly, the evaluation of valgus deformity was performed by a clinical method which raises concerns about its reliability and reproducibility. However, the clinical method of measuring the knee angle is validated [31], widely accepted, easily reproducible and is inexpensive and radiation-free [34]. Moreover, it was performed by an experienced paediatric orthopaedic surgeon using fixed bony points to calculate the TF angle and the IM distance. Additionally, the palpation of bony landmarks is practical and easy, diminishing the possibility of an erroneous measurement even in obese children [34]. Third, the results are not accompanied by supportive analysis of biological data. Finally, the small number of dizygotic twins did not allow us to draw definite conclusions.

In the present study, GP prevalence is high, corresponding to 29.88% of children aged 3–7 years. The reported prevalence differs between various surveys. Evans et al. estimated that the frequency of GPs was 36.9% in children 4–6 years of age [3]. In a Mediterranean population of children 3–12 years old the frequency of the disorder was 24.5% [4], while Oster found that 15% of school children have occasional limb pain [35]. In a very large British cohort study, but without applying Peterson’s criteria for GP assessment, the referred prevalence was 21.4% [14]. Overall, their prevalence varies from 2.6% to 49.4% [36]. Differences not only in sample size or age range and definition criteria but also in ethnicity features may explain this discordance.

Anatomical or mechanical factors were always considered in the research of GPs [2,35]. In our study, the severity of genu valgum (increased IM distance and TF angle) was statistically associated with the development of GPs in children, though it was not correlated with their frequency or intensity. Additionally, increased IM distance and TF angle were strongly related with GPs in dizygotic twins. As we assume that dizygotic twins stem from the same gene pool and are exposed to similar environmental factors [37], the knee deformity may have a critical contribution in the appearance of the pains. This finding is in agreement with Brenning’s observation that anatomical deformities which cause mechanical instability of the lower limbs including scoliosis, pes planus or genu valgum are closely related with GPs [38]. This is also supported by the study of Evans [6] where the correction of the lower extremity malalignment with shoe inserts reduces the frequency and the severity of GPs. Furthermore, the application of customized foot orthoses that control overpronation and decrease the secondary genu valgum of the knee joint in children with overpronated feet led to significant improvement in pain degree and frequency after 1 and 3 months [39]. On the contrary, in the study of Evans and Scatter, although the navicular height was weakly correlated with foot functional indexes, the overall foot posture examination measurements did not reveal any statistical significance with GPs [40].

Perinatal risk factors were closely related not only with the appearance of GPs but also with their frequency and magnitude. Specifically, the risk of GP appearance was statistically increased when the birth weight of the child was lower than 3000 g. Additionally, the possibility of GP development was increased in children with birth head circumference smaller than 33 cm. In children that present GPs, the possibilities of nocturnal waking or crying and suffering from pain in both legs was also increased in the aforementioned groups. Several studies have shown that whole-body bone mineral content (BMC) correlates with gestational age, body length and body weight. Moreover, growth of head circumference in infants was found to be synchronized with long bone growth and integrity [41]. Research data suggest that there was a decrease in bone mineral density (BMD) in animals with intrauterine growth restriction [42]. These animals also had reduced serum concentrations of vitamin D during their first 20 weeks of life which is in line with the observations of Monangi et al. [43], whereby the odds of low serum concentrations of vitamin D was increased 2.6-fold in infants born less than 28 weeks postmenstrual age. Newborns with low birth weight also had lower BMC [44]. Similarly, postnatal measurements with quantitative ultrasound confirm that the values of tibial speed of sound are affected by a short gestation period [45]. This is consistent with the results of Kurl et al. [17] which demonstrated that remarkably lower BMC and BMD were detected in preterm infants than the term group. It is noteworthy that adolescents with idiopathic short stature had a reduction in their BMD [46]. Wang et al. reported that very low birth weight infants had reduced bone mass at ages 5 to 10 years, even after adjustment for height, lean mass, or bone area [47], whereas in the same study, short gestation period was correlated with unfavorable skeletal health among prepubertal boys [47]. Predicated on the concept that vitamin D deficiency [12,13,14,15,16] and BMD [8] are key factors for the development of GPs, further research may reveal if the implication of perinatal risk factors in the bone metabolism process results in the onset of the disorder.

Contrariwise, antenatal corticosteroid treatment, increased maternal age and maternal smoking during pregnancy were not predictive of GPs. Despite the fact that antenatal administration of corticosteroids was not a contributing factor to the development of GPs, the frequency and the intensity of GPs were elevated in the children who had been exposed to dexamethasone. In children that present GPs, the use of corticosteroids was closely related with nocturnal waking or crying and suffering from pain in both legs. Furthermore, our data indicates that in these children the frequency of GP episodes was as high as once per week. Although corticosteroids are implicated in bone metabolism, many studies have failed to show distinct differences in BMC values between dexamethasone infants and controls [18,48,49]. Furthermore, Korakaki et al. reported that bone formation serum marker concentration was unaffected or slightly reduced at birth in neonates of mothers treated with corticosteroids compared to control subjects, while the bone resorption markers did not differ among the two groups [24]. Furthermore, the bone collagen markers in the newborns exposed to dexamethasone were restored during the first ten days of life [24]. Based on the above findings, we can hypothesize that the antenatal administration of corticosteroids does not affect the onset of GPs due to the minor impact of dexamethasone on bone turnover in infants.

## 5. Conclusions

The findings of the present study demonstrate that perinatal risk factors can be predictive of the development of growing pains. The close association of gestational age, head circumference and birth length or weight with bone turnover could support the bone strength theory for this disorder, indicating to clinicians a detailed bone monitoring of this subgroup. Furthermore, our results also support the anatomical theory as the degree of genu valgum has a significant effect on the development of GPs, leading to the notion that an orthotic correction of severe valgus deformity may be an efficient intervention for the prevention or the treatment of this condition.

## Figures and Tables

**Table 1:** Demographic characteristics of the study population.

	Total Sample (*n* = 261)				With Growing Pains (*n* = 78)				Without Growing Pains (*n* = 183)				Dizygotic Twins (*n* = 20)			
	Mean	Min	Max	SD	Mean	Min	Max	SD	Mean	Min	Max	SD	Mean	Min	Max	SD
Age (years)	5.07	3.00	7.50	1.22	5.15	3.00	7.00	1.21	5.04	3.00	7.50	1.22	4.95	3.00	7.00	1.10
Weight (kg)	21.2	13.0	50.0	5.42	22.1	13.5	50.0	6.59	20.9	13.0	38.5	4.81	19.5	13.5	30.0	4.35
Height (cm)	114	91.0	139	9.58	115	97.5	139	9.58	113	91.0	134	9.50	111	97.5	127	7.62
BMI (kg/m^2^)	16.2	11.4	27.9	3.53	16.2	11.4	27.9	2.64	10.2	11.4	22.7	1.97	15.7	12.5	19.6	1.83

SD: standard deviation.

**Table 2:** Descriptive clinical features and statistical analysis of the study participants.

Clinical Characteristics	With Growing Pains						Without Growing Pains						Statistical Significance		
	Mean	Min	Max	SD	95% CI		Mean	Min	Max	SD	95% CI		*t*	*Z*	*p* Value
					Lower Bound	Upper Bound					Lower Bound	Upper Bound			
Genu valgum															
TF angle (degrees)	5.30	0.00	9.00	2.30	4.49	6.12	2.90	0.00	4.00	1.15	2.34	3.45		−7.07	<0.01
IM distance (cm)	3.32	0.00	6.00	1.54	2.77	3.86	1.34	0.00	3.00	0.96	0.88	1.80	−9.61		<0.01
Perinatal factors															
Gestational age (weeks)	36.8	28.0	40.0	2.49	35.9	37.6	37.7	34.0	40.0	1.63	36.9	38.5		−4.44	<0.01
Corticosteroid treatment (doses)	1.91	1.00	4.00	0.77	1.64	2.18	1.84	1.00	4.00	0.83	1.44	2.24		−0.43	0.57
Apgar score	8.42	6.00	10.0	1.12	7.88	8.96	8.76	6.00	10.0	1.15	8.35	9.16		−2.36	0.02
Birth length (cm)	47.9	40.0	54.0	2.78	47.0	48.9	49.9	45.0	54.0	2.09	48.9	50.9		−5.18	<0.01
Birth weight (g)	2727	1395	3880	579	2522	2932	2983	1650	4040	489	2747	3219	3.66		<0.01
Head circumference (cm)	33.2	28.0	37.5	1.90	32.5	33.8	34.4	31.4	38.0	1.57	33.6	35.2	3.51		<0.01
Maternal age (years)	31.7	21.0	40.0	4.60	30.1	33.4	33.2	26.0	41.0	3.72	31.4	35.0	−0.12		0.91

CI: confidence interval; TF angle: tibiopfemoral angle; IM distance: intermalleolar distance.

**Table 3:** Growing pains and their association with perinatal factors.

Variable	Present Pains (*n* = 78)	Do Not Present Pains (*n* = 183)	Total (*n* = 261)	*p* Value
*Birth weight*				
<3000 g	*n* = 42 (56.8%)	*n* = 32 (43.2%)	*n* = 74	<0.01
>3000 g	*n* = 36 (19.3%)	*n* = 151 (80.7%)	*n* = 187	
*Head circumference*				
<33 cm	*n* = 25 (59.5%)	*n* = 17 (40.5%)	*n* = 42	<0.01
>33 cm	*n* = 53 (24.2%)	*n* = 166 (75.8%)	*n* = 219	
*Maternal smoking*				
Non-smokers	*n* = 63 (27.8%)	*n* = 164 (72.2%)	*n* = 227	0.05
Smokers	*n* = 15 (44.1%)	*n* = 19 (55.9%)	*n* = 34	

**Table 4:** Results of statistically significant factors for the development of growing pains derived after logistic regression analysis.

Risk Factors	B	SE	Wald	95% CI		*p* Value
				Lower	Upper	
Sex	−0.86	0.37	5.33	0.21	0.88	0.02
Children’s age (years)	0.31	0.15	4.21	1.02	1.84	0.04
IM distance (cm)	0.99	0.15	45.5	2.02	3.61	<0.01
Gestational age (weeks)	1.95	0.50	15.3	0.71	1.26	<0.01
Apgar score	−0.33	0.22	2.14	0.46	1.12	0.14
Maternal age (years)	−0.02	0.04	0.29	0.91	1.06	0.54
Smoking	0.33	0.56	0.34	0.46	4.18	0.56

SE: standard error, Wald: Wald chi-square test.

**Table 5:** Comparison of growing pains intensity with the perinatal factors’ characteristics and knock-knee severity of the affected children.

Type of Pain	Mean	SD	SE	*t*	*p* Value
*Nocturnal waking* (*n* = 42)					
IM distance (cm)	3.43	1.36	0.21	−0.43	0.67
Birth length (cm)	48.5	2.81	0.43	3.47	<0.01
Head circumference (cm)	33.4	1.89	0.29	3.35	<0.01
Maternal age (years)	31.6	3.75	0.58	−1.47	0.15
*Crying* (*n* = 36)					
IM distance (cm)	3.47	1.43	0.24	−0.63	0.53
Birth length (cm)	48.6	3.05	0.51	2.37	0.02
Head circumference (cm)	33.4	1.91	0.32	2.93	0.02
Maternal age (years)	31.7	3.74	0.62	−1.52	0.14
*Pains in both legs* (*n* = 51)					
IM distance (cm)	3.32	1.45	0.20	0.38	0.71
Birth length (cm)	48.6	2.57	0.36	4.47	<0.01
Head circumference (cm)	33.4	1.57	0.22	5.30	<0.01
Maternal age (years)	31.7	4.67	0.65	−2.46	0.01

**Table 6:** Statistical analysis for the relationship between types of growing pains, perinatal factors and use of corticosteroids.

Type of Pain	Birth Length		*p* Value	Birth Weight		*p* Value	Head Circumference		*p* Value	Use of Corticosteroids		*p* Value	Total
	<50 cm	>50 cm		<3000 g	>3000 g		<33 cm	>33 cm		Yes	No		
*Nocturnal waking*	*n* = 32 (76.2%)	*n* = 10 (23.8%)	0.05	*n* = 31 (73.8%)	*n* = 11 (26.2%)	<0.01	*n* = 22 (52.4%)	*n* = 20 (47.6%)	<0.01	*n* = 27 (64.3%)	*n* = 15 (35.7%)	<0.01	*n* = 42
*Crying*	*n* = 26 (72.2%)	*n* = 10 (27.8%)	0.47	*n* = 28 (77.8%)	*n* = 8 (22.2%)	<0.01	*n* = 19 (52.8%)	*n* = 17 (47.2%)	<0.01	*n* = 21 (58.3%)	*n* = 15 (41.7%)	<0.01	*n* = 36
*Pains in both legs*	*n* = 40 (78.4%)	*n* = 11 (21.6%)	<0.01	*n* = 34 (66.7%)	*n* = 17 (33.3%)	<0.01	*n* = 23 (45.1%)	*n* = 28 (54.9%)	<0.01	*n* = 28 (54.9%)	*n* = 23 (45.1%)	<0.01	*n* = 51

**Table 7:** Comparison of the frequency of growing pains appearance with perinatal factors’ characteristics and knock-knee severity of the affected children.

Frequency of Pain	Mean	SD	SE	Min	Max	*F* Value	*p* Value
*Weekly* (*n* = 19)							
IM distance (cm)	3.37	1.55	0.36	0.00	5.50	0.25	0.86
Birth length (cm)	48.7	2.31	0.53	45.0	53.0	1.63	0.19
Head circumference (cm)	33.5	1.75	0.40	31.0	37.0	5.22	<0.01
Maternal age (years)	31.8	3.49	0.80	27.0	37.0	0.90	0.45
*Monthly* (*n* = 33)							
IM distance (cm)	3.33	1.27	0.22	1.00	6.00		
Birth length (cm)	49.1	3.05	0.53	40.0	54.0		
Head circumference (cm)	33.5	1.74	0.30	28.0	37.0		
Maternal age (years)	31.3	4.38	0.76	23.0	39.0		
*Every three months* (*n* = 19)							
IM distance (cm)	3.26	1.48	0.34	0.00	6.00		
Birth length (cm)	50.2	1.56	0.36	48.0	53.0		
Head circumference (cm)	35.0	0.97	0.22	33.5	37.5		
Maternal age (years)	30.1	4.99	1.15	21.0	40.0		
*Every six months* (*n* = 7)							
IM distance (cm)	3.79	1.38	0.52	2.00	6.00		
Birth length (cm)	50.3	1.05	0.40	49.3	52.0		
Head circumference (cm)	34.7	0.76	0.30	33.5	35.8		
Maternal age (years)	29.1	5.43	2.05	24.0	40.0		

**Table 8:** Association between the frequency of growing pains, birth weight and perinatal corticosteroid administration.

Frequency of Pain	Birth Weight		*p* Value	Administration of Corticosteroids		*p* Value	Total
	<3000 g	>3000 g	<0.01	With Corticosteroids	Without Corticosteroids	<0.01	
Weekly	*n* = 14 (73.7%)	*n* = 5 (26.3%)		*n* = 10 (52.6%)	*n* = 9 (47.4%)		*n* = 19
Monthly	*n* = 21 (63.6%)	*n* = 12 (36.4%)		*n* = 17 (51.5%)	*n* = 16 (48.5%)		*n* = 33
Every three months	*n* = 4 (21.1%)	*n* = 12 (78.9%)		*n* = 6 (31.6%)	*n* = 13 (68.4%)		*n* = 19
Every six months	*n* = 3 (42.9%)	*n* = 4 (57.1%)		*n* = 0 (0%)	*n* = 7 (100%)		*n* = 7

**Table 9:** Results derived after the Spearman correlation test examined the association between the FACES scale and the risk factors for the development of growing pains.

Risk Factors	FACES Scale		
	*n*	Correlation Coefficient	*p* Value
TF angle (degrees)	78	0.07	0.52
IM distance (cm)	78	0.11	0.35
Gestational age (weeks)	78	−0.32	0.01
Administration of corticosteroids (doses)	33	0.32	0.07
Apgar score	78	0.03	0.78
Birth length (cm)	78	−0.48	<0.01
Birth weight (g)	78	−0.49	<0.01
Head circumference (cm)	78	−0.43	<0.01
Maternal age (years)	78	0.03	0.78

**Table 10:** Statistical analysis of the dizygotic twins’ clinical characteristics.

Clinical Characteristics	Present Growing Pains (*n* = 9)				Do Not Present Growing Pains (*n* = 11)				Statistical Significance	
	Mean	Min	Max	SD	Mean	Min	Max	SD	Z	*p* Value
Current weight (kg)	19.0	13.5	30.0	5.46	19.9	14.0	26.0	3.42	−0.10	0.92
Current height (cm)	109	97.5	127	9.05	112	102	123	6.17	−1.66	0.10
Current BMI (kg/m^2^)	15.8	12.5	19.6	2.30	15.6	12.5	17.2	1.45	−0.66	0.51
TF angle (degrees)	6.22	3.00	8.00	1.48	2.91	1.00	4.00	1.04	−2.33	0.02
IM distance (cm)	3.89	2.50	5.50	0.96	1.36	0.00	3.00	0.92	−2.41	0.02
Birth length (cm)	47.5	42.0	52.0	2.89	49.1	45.0	51.0	2.23	−1.07	0.28
Birth weight (g)	2444	1600	2910	417	2786	1650	3260	471	−1.23	0.22
Head circumference (cm)	33.7	31.5	37.5	1.79	33.7	31.4	36.5	1.57	−0.60	0.57
Apgar score	7.89	6.00	10.0	1.62	7.91	6.00	9.00	1.14	−0.14	0.89
